# Exogenous nitric oxide decreases brain vascular inflammation, leakage and venular resistance during *Plasmodium berghei* ANKA infection in mice

**DOI:** 10.1186/1742-2094-8-66

**Published:** 2011-06-07

**Authors:** Graziela M Zanini, Pedro Cabrales, Wisam Barkho, John A Frangos, Leonardo JM Carvalho

**Affiliations:** 1 La Jolla Bioengineering Institute, San Diego, CA, USA; 2 Parasitology Service, Instituto de Pesquisas Clinicas Evandro Chagas, Fiocruz, Rio de Janeiro, Brazil; 3 Department of Bioengineering, University of California, San Diego, CA, USA

## Abstract

**Background:**

Cerebral malaria (CM) is a lethal complication of Plasmodium falciparum infections. In the Plasmodium berghei ANKA (PbA) murine model, CM is associated with marked brain inflammation, increased expression of endothelial cell adhesion molecules and leukocyte and platelet accumulation in brain vessels, causing vascular occlusion and decreased blood flow, damaging the endothelium and leading to blood-brain barrier breakdown, leakage and hemorrhages. Exogenous nitric oxide (NO) administration largely prevents the syndrome. Here we evaluated whether the mechanism of action of NO in preventing murine CM is related to its anti-inflammatory properties and to protection of the endothelium.

**Methods:**

C57Bl/6 mice infected with PbA were treated twice a day with saline or dipropylenetriamineNONOate (DPTA-NO). Endothelial cell adhesion molecule (ICAM-1, VCAM, E- and P-selectin) expression in brain tissue on day 6 of infection was assessed in both groups by western blot. For intravital microscopy studies, DPTA-NO-treated and saline-treated mice with a previously implanted closed cranial window were injected with albumin-FITC, anti-CD45-TxR and anti-CD41-FITC antibodies on day 6 of infection for quantification of albumin leakage, leukocyte and platelet adherence in pial vessels.

**Results:**

PbA-infected mice treated with the NO-donor DPTA-NO showed decreased expression of ICAM-1 and P-selectin, but not VCAM-1, in the brain, compared to saline-treated mice. DPTA-NO treatment also decreased the number of adherent leukocytes and platelets in pial vessels, particularly in venules 30-50 μm in diameter, decreased inflammatory vascular resistance and prevented the occurrence of arteriolar and venular albumin leakage observed in saline-treated PbA-infected mice, as assessed by intravital microscopy.

**Conclusions:**

These results indicate that the protective effect of exogenous NO on murine CM is associated with decreased brain vascular expression of inflammatory markers resulting in attenuated endothelial junction damage and facilitating blood flow.

## Introduction

Cerebral malaria (CM), a complication of malaria infection by Plasmodium falciparum, is a leading cause of mortality and neurological impairment in endemic areas, with an estimated 1 million deaths every year [1]. In the murine model of CM by P. berghei ANKA (PbA), the neurological syndrome is associated with several indicators of severe vasculopathy and endothelial dysfunction, whose pathogenesis is complex and involves a systemic inflammatory response with activation of CD4+ and CD8+ T cells, macrophages, platelets, release of several pro-inflammatory cytokines such as IFN-γ, TNF-α and LTA [2-8] as well as depletion of nitric oxide (NO) [9] and increased expression of endothelin-1 and other vasoactive elements [10,11]. These events cause the vascular beds in the brain and other organs to upregulate the expression of a number of endothelial cell adhesion molecules (eCAMs), including ICAM-1, VCAM-1 and P-selectin [12]. The activated endothelium facilitates the recruitment of different cell types, including monocytes, lymphocytes, platelets and pRBCs, which interact with the eCAMs, roll and adhere, accumulating, and ultimately plugging the brain vessels and causing blood flow disturbances or even complete vascular blockades [13]. Interaction with adherent cells can also result in endothelial cell dysfunction and damage through direct contact or the release of active compounds such as cytokines and perforin, leading to BBB disruption, with leakage of plasma contents into the brain parenchyma which can induce neurotoxicity or contribute to brain edema [14-16]. A damaged vessel may eventually rupture causing the disseminated hemorrhages commonly observed in murine CM [17,18].

Endothelial dysfunction by different mechanisms such as NO depletion can contribute to vascular inflammation. It has been shown that low NO bioavailability or NO inhibition can induce the expression of eCAMs [19-21], platelet activation and adhesion to endothelial cells [22,23] and increase baseline leukocyte rolling and adherence [24,25], and administration of NO donors inhibits endothelial activation [26]. Low NO bioavailability is a major player in vascular inflammation commonly observed in hemolytic syndromes such as sickle cell crisis, in which acellular hemoglobin acts as a strong NO scavenger [27]. Murine CM is associated with low NO bioavailability due largely to NO-scavenging by plasma hemoglobin, and exogenous supplementation of NO to PbA-infected mice largely prevents CM [9]. We have recently shown that this protection is associated with improved cerebral microcirculatory function, as NO supplementation prevented pial vasoconstriction and attenuated the decrease in pial blood flow in PbA-infected mice [18].

In the present study, we show that NO supplementation acts by decreasing eCAM expression in the brain leading to decreased adherence of leukocytes and platelets in pial vessels and preventing vascular leakage in arterioles and venules.

## Methods

### Parasite, infection and NO-donor treatment

Animal handling and care followed the NIH Guide for Care and Use of Laboratory Animals. All protocols were approved by the La Jolla Bioengineering Institutional Animal Care and Use Committee. Eight to 12-week old C57Bl/6 (Jackson Laboratories, ME) were inoculated intraperitoneally (IP) with 1 × 10^6 ^Plasmodium berghei ANKA parasites expressing the green fluorescent protein (PbA-GFP, a donation from the Malaria Research and Reference Reagent Resource Center - MR4, Manassas, VA; deposited by CJ Janse and AP Waters; MR4 number: MRA-865). Parasitemia, body weight, rectal temperature and clinical status (using six simple tests adapted from the SHIRPA protocol, as previously described [18], were monitored daily from day 4. Parasitemia was checked by flow cytometry by detecting the number of fluorescent GFP-expressing pRBCs in relation to 10,000 RBCs. CM was defined as the presentation of one or more of the following clinical signs of neurological involvement: ataxia, limb paralysis, poor righting reflex, seizures, roll-over, coma. PbA-infected mice were treated with either saline or dipropylenetriamine NONOate (DPTA-NO, Cayman Chemical, Ann Arbor, MI) 1 mg/mouse, intraperitoneally (IP), twice a day starting on day 0 of infection.

### Brain endothelial cell adhesion molecule expression quantification

On day 6 of infection, saline-treated and DPTA-NO-treated PbA-infected mice, as well as uninfected control mice, were euthanized with 100 mg/kg of euthasol IP, perfused with saline and the brains collected and frozen at -80°C until processing. Brains were defrosted and homogenized in lysis buffer (50 mM Tris pH7.5, 125 mM NaCl, 60 mM octylglucoside, 2 mM sodium vanadate and protease inhibitor cocktail tablets (Complete, Roche), on ice for 30 min than centrifuged at 14.000 g for 20 min, the supernatant was diluted and proteins were resolved by sodium dodecyl-sulphate polyacrylamide gel electrophoresis and then transferred onto nitrocellulose membranes (Milipore). Membranes were blocked and probed with antibodies to intercellular cell adhesion molecule-1 (ICAM-1), vascular cell adhesion molecule-1 (VCAM-1), P-selectin or E-selectin (all from BD Biosciences). Detection was performed with anti-IgG secondary antibodies conjugated to peroxidase and reaction was developed with Super Signal West Pico Chemiluminescence Substrate (Thermo Scientific). After development, antibodies were unbound using Restore Western Blot Stripping buffer (Thermo Scientific), re-incubated with antibodies to GAPDH (V-18) (Santa Cruz) and reaction was developed by chemiluminescence. The band intensities were determined after scanning the radiographic films and image analysis using ImageJ (NIH) software, and eCAM expression was normalized in relation to GAPDH expression.

### Quantification of brain leukocyte and platelet adherence and vascular leakage using intravital microscopy

The closed cranial window model was used as previously described [13,28]. After the implantation of the cranial window, mice rested for 2-4 weeks before starting the experiment. On the day before infection, a panoramic picture of the vascular network under the window was taken and, with the mouse under light isoflurane anesthesia, the pial vessels were checked in the intravital microscope and 12 arterioles and venules were randomly selected and marked in the panoramic picture, and diameters measured using an image shear device (Image Shear, Vista Electronics, San Diego, CA). Mice were inoculated with PbA the next day, followed up and treated with either saline or DPTA-NO as described above. On day 6 of infection, a cocktail containing albumin-FITC (Sigma, St Louis, MO; 50 μg), anti-CD45-TxR antibodies (CalTag, Carlsbad, CA; 4 μg) and anti-CD41-FITC antibodies (CalTag, Carlsbad, CA; 4 μg) was intravenously infused through the tail vein (final volume: 75 μL). The mouse was then lightly anesthetized with isoflurane (4% for induction, 1-2% for maintenance) and put on a stereotaxic frame with the head gently held with ear bars. A panoramic picture of the vessels under the window was taken and the mouse was transferred to the intravital microscope stage. Body temperature, measured pre-anesthesia, was maintained with a heating pad. Using water-immersion objectives (20X), blood vessel images were captured (COHU 4815, San Diego, CA) and recorded on video-tape. Diameter measurements of 6-10 pial venules and 2-6 pial arterioles previously selected as described above were performed in each animal. Fifteen minutes after injection of the fluorescent-labeled markers, green fluorescence (518 nm) emitted by albumin-FITC, GFP (PbA pRBC) and anti-CD41-FITC antibodies (platelets) was captured using an ALPHA Vivid: XF100-2 (Omega Optical, Brattleboro, VT), and red fluorescence (615 nm) emitted by anti-CD45-TxR antibodies was exited and captured with a Vivid Standard: XF42. Adherence was defined as cells remaining static for 30 seconds. For each selected vessel, leukocytes and platelets were quantified in a 100 μm-long section. The pre-selection of vessels on day 0 to perform the measurements was intended to avoid bias in the quantifications, as different vessels may be heterogeneously affected by leukocyte and platelet adherence. Vascular leakage was measured by quantifying the fluorescence emitted by albumin-FITC (Molecular Probes, Eugene, OR) inside the vessel and in the surrounding tissue, using ALPHA Vivid: XF100-2 (Exciter: XF1073; Dichroic: XF2010; Emitter: XF3084, Omega Filters Brattleboro, VT). The albumin-FITC was injected into the tail vein and 15 min after infusion the fluorescence emission signals were recorded using a photomultiplier (Hamamatsu R928, Tokyo, Japan). Results of leakage are expressed as the ratio of albumin-FITC-derived fluorescence intensity between the tissue and the vessel. Higher ratios correspond to higher leakage. Inflammation effects on vascular resistance were calculated by mathematically using Darcy's law and Hagen-Poiseuille equation relative to without inflammation. These estimations were calculated by each microvessel using the ratio between the microvessel with the volumes occupied by adhered leukocytes relative to without leukocytes and approximating the pressure drop through a non-variable length cylindrical pipe as a function of the diameter to the power of four (area square).

### Statistical analysis

Statistical analyses were performed using analysis of variance (ANOVA) with Dunnett's post-hoc analysis to compare eCAM expression, leukocyte and platelet adherence and vascular leakage in uninfected, saline-treated and DPTA-NO-treated PbA-infected mice. Correlations were established using the Spearmann rank test. All statistics were performed using the Graphpad Prism software (GraphPad Software Inc., La Jolla, CA). A p value < 0.05 was considered significant.

## Results

### DPTA-NO treatment decreases ICAM-1 and P-selectin, but not VCAM-1, expression in the brain of PbA-infected mice

**Figure 1:**
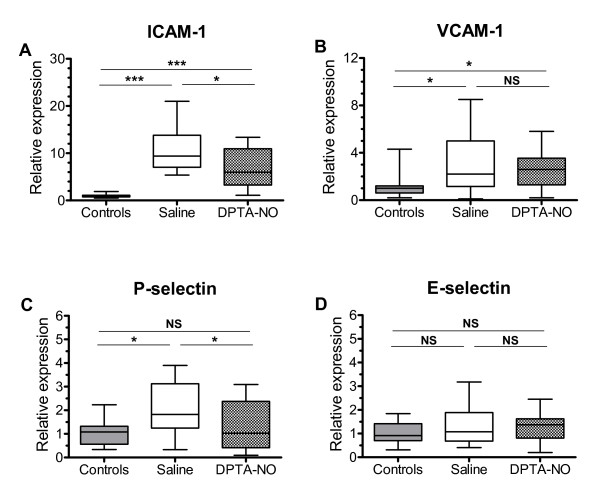
**Nitric oxide decreases cell adhesion molecule expression in the brain during Plasmodium berghei ANKA infection**. Expression levels of ICAM-1, VCAM-1, P-selectin and E-selectin in the brain of uninfected and PbA-infected saline-treated and DPTA-NO-treated mice (day 6 of infection). Expression levels were normalized in relation to GAPDH expression. Saline-treated mice showed increased expression of ICAM-1, VCAM-1 and P-selectin, but not E-selectin. DPTA-NO treatment significantly decreased ICAM-1 and P-selectin, but not VCAM-1, expression. *: p < 0.05; **: p < 0.01; ***: p < 0.001.

In the eCAM expression studies, 79% of the saline-treated mice (n = 14) showed clinical signs of CM on day 6 of infection, against 29% of the DPTA-NO-treated mice (n = 14). All mice were euthanized regardless of CM expression and brains were collected. The course of parasitemia was similar in both groups (saline: 10.8 ± 0.58%; DPTA-NO: 10.2 ± 0.65%, day 6 of infection). Saline-treated PbA-infected mice showed increased expression of ICAM-1, VCAM-1 and P-selectin, but not E-selectin (Figure 1). ICAM-1 expression was increased nearly 10-fold, whereas VCAM-1 and P-selectin expression was increased about two-fold in relation to uninfected controls. DPTA-NO treatment caused significant reductions in ICAM-1 and P-selectin expression, but did not significantly modify VCAM-1 expression (Figure 1).

### DPTA-NO treatment decreases leukocyte and platelet sequestration in the brain

**Figure 2:**
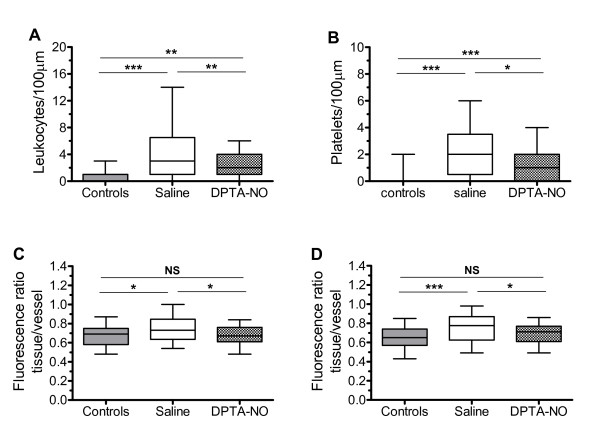
**Nitric oxide decreases leukocyte and platelet adherence and vascular leakage during Plasmodium berghei ANKA infection**. Mean number of adherent leukocytes (A) and platelets (B) in pial venules, and albumin leakage in arterioles (C) and venules (D) of saline-treated (n = 6) and DPTA-NO-treated (n = 6) PbA-infected mice on day 6 of infection, and in uninfected control mice (n = 5). A total of 6-8 venules and 4-6 arterioles were analyzed per mouse. Saline-treated mice showed increased number of adherent leukocytes and platelets, as well as increased vascular permeability, and DPTA-NO treatment markedly inhibited adherence and leakage. **: p < 0.01; ***: p < 0.001; NS: non-significant.

Leukocyte/platelet adherence and albumin-FITC leakage were evaluated in pial arterioles and venules ranging from 20 to 50 μm in diameter using intravital microscopy. Saline-treated PbA-infected mice showed increased number of sequestered adherent leukocytes and platelets in pial venules on day 6 of infection (Figure 2A,B). Treatment with DPTA-NO significantly reduced the number of both leukocytes and platelets in pial vessels.

### DPTA-NO treatment prevents albumin leakage in pial arterioles and venules

Mice with CM show breakdown of the blood-brain barrier, as generally observed by whole brain leakage of Evans blue dye [29]. Evaluation of albumin leakage in individual vessels by intravital microscopy revealed that leakage occurred in both arterioles and venules and was prevented by treatment with DPTA-NO (Figure 2C,D).

### Adherent leukocytes and platelets, but not leakage, co-localize

We asked whether vessel size would affect leukocyte/platelet adherence and intensity of leakage in saline-treated PbA-infected mice, and whether venules presenting higher numbers of adherent leukocytes would also present higher platelet adherence and more intense leakage. Leukocyte (r^2 ^= 0.21) and platelet (r^2 ^= 0.15) adherence, but not albumin leakage (r^2 ^= 0.03), were significantly correlated with increasing venular diameters. Vessels with more leukocytes also presented more platelets, this correlation being also present in DPTA-NO-treated mice despite the lower numbers of adherent cells. Platelet adherence (r^2 ^= 0.49), but not leakage (r^2 ^= 0.01), was significantly correlated with leukocyte adherence in venules. No correlation was found between leukocyte or platelet adherence and leakage (r^2 ^= 0.06).

### Effect of exogenous NO on leukocyte and platelet accumulation differs according to venular diameter

**Figure 3:**
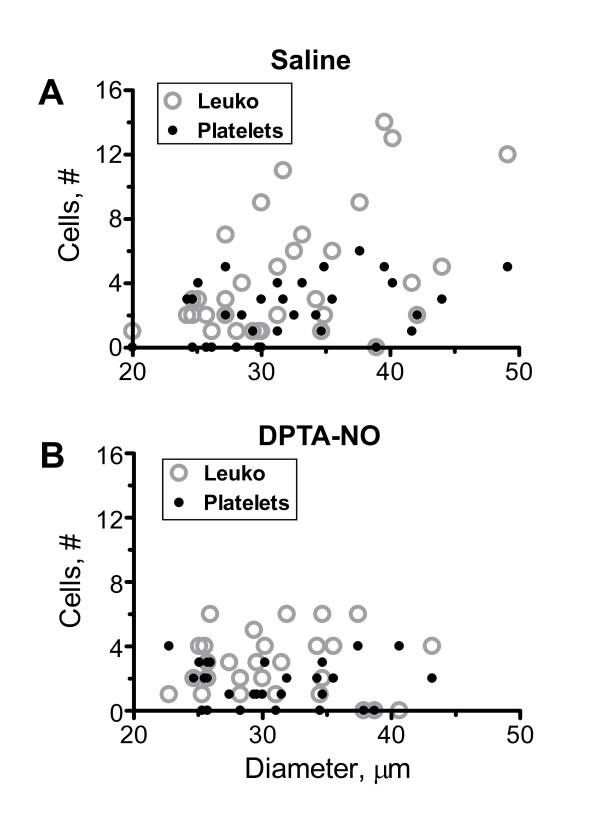
**Effect of DPTA-NO treatment on leukocyte and platelet adherence during PbA infection**. (A): Leukocyte and platelet adherence significantly correlate with increasing venular diameters in saline-treated (A) but not in DPTA-NO-treated (B) PbA-infected mice on day 6 of infection.

DPTA-NO treatment affected the distribution of adherent leukocytes at different microvessel diameters. While in saline-treated animals the number of adherent leukocytes increased with increasing venular diameter, in DPTA-NO-treated mice the distribution of adherent leukocytes was relatively homogeneous in microvessels of different diameters (Figure 3A,B). These results suggest that DPTA-NO largely inhibited leukocyte accumulation in vessels larger, but not smaller, than 30 μm.

### Decrease in leukocyte accumulation by exogenous NO affect vascular resistance

**Figure 4:**
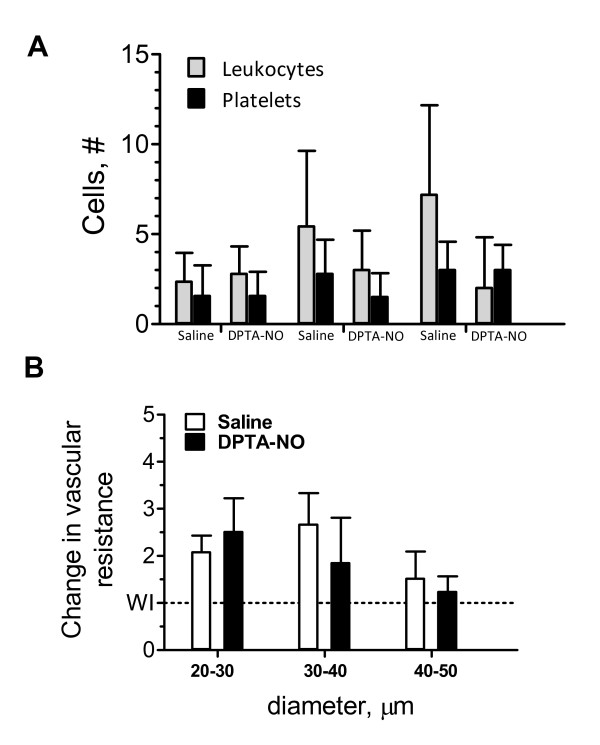
**Effect of DPTA-NO treatment on venular resistance to blood flow on day 6 of infection**. Venules were categorized in three brackets according to diameter: 20-30 μm, 30-40 μm and 40-50 μm. (A): number of adherent leukocytes and platelets per category: accumulation of leukocytes and platelets increases in the higher diameter brackets in saline-treated but not in DPTA-NO-treated mice. (B): Venular resistance to blood flow is increased in all brackets in saline-treated PbA-infected mice in relation to vessels without inflammation (WI - dashed line), particularly in the 30-40 μm range (P < 0.05). DPTA-NO treatment has no effect on venular resistance in smaller (20-30 μm) venules but significantly decreases resistance in larger (30-40 μm) venules. Although the number of leukocytes is higher in venules 40-50 μm in saline-treated PbA-infected mice, the effect on resistance to blood flow is minor. Data in (A) and (B) are the mean ± standard deviation.

To analyze the effect of leukocyte accumulation on venular resistance to blood flow, venules were categorized in three groups based on diameter: 20-30 μm, 30-40 μm and 40-50 μm. Figure 4A shows the mean number of leukocytes and platelets per category. In venules of saline-treated PbA-infected mice, leukocyte accumulation resulted in increased resistance to blood flow, particularly in the range of 20-40 μm in diameter (Figure 4B). Due to larger diameters the degree of leukocyte accumulation in vessels 40-50 μm-diameter did not substantially affect vascular resistance. DPTA-NO treatment had little effect upon leukocyte accumulation in smaller venules (20-30 μm in diameter) and therefore its effect on decreasing vascular resistance was felt only in vessels in the 30-40 μm-diameter range. Because hydrostatic pressure is lower in larger venules, the reduction of vascular resistance in larger venules is expected to increase venous return in DPTA-NO-treated mice.

## Discussion

Murine cerebral malaria is associated with increased expression of eCAMs as well as leukocyte and platelet accumulation in brain vessels leading to microvascular damage and BBB breakdown [8,30]. Here we show that exogenous NO supplementation attenuated, although it did not prevent, brain vascular inflammation (eCAM expression, leukocyte and platelet accumulation) and its consequences such as vascular leakage. We have previously shown that exogenous NO supplementation ameliorated pial blood flow, vasoconstriction and the occurrence of brain hemorrhages [18]. Overall, these results indicate that low NO bioavailability has a role in the microvascular dysfunction observed during murine CM [13] and that exogenous NO can help to protect the vasculature, attenuating endothelial damage and dysfunction. The only partial effects of DPTA-NO treatment on inflammatory markers and on brain hemodynamics suggest that NO supplementation provides a subtle beneficial effect on CM pathogenesis, which for a significant number of animals can be enough to prevent death by CM, rather than an unmistakable modification of disease history. In addition, the overlap between treated and untreated mice in several of the measured parameters might suggest that NO-mediated mechanisms other than the ones reported here could be responsible for the clinical effect.

Studies of eCAM expression in human and murine CM consistently show an increased expression of ICAM-1, whereas the results for other eCAMs are variable. In cases of fatal human CM, the picture that emerges is of marked increases in ICAM-1, significant but less pronounced increases in VCAM-1 and E-selectin and no changes in P-selectin expression in the brain [31-33]. In murine CM, high expression of ICAM-1 in the brain is consistently observed [12,34,35] and VCAM-1 expression has been shown to be also markedly increased in brain and retinal vessels [12,35]. P-selectin was shown to be increased whereas no change in E-selectin was reported on day 6 of infection [12,36]. Similarly, in the present study, we found a marked increase in ICAM-1 expression in the brain on day 6 of infection, VCAM-1 and P-selectin had more modest increases and E-selectin expression was not significantly different in uninfected and saline-treated mice. ICAM-1, indeed, seems to play a relevant role in CM, as not only its expression in brain is consistently markedly increased, but also ICAM-1 gene knockout mice are protected from CM development [37,38]. The roles of VCAM-1, E- and P-selectin are less clear. Increased expression of E- and P-selectin seems to be less intense in the brain as compared to other organs and tissues during PbA infection and also sepsis [12,39]. Whether the lower magnitude of expression of selectins in the brain is relevant for CM development is debatable. P-selectin gene knockout did not change leukocyte accumulation in the brain during murine CM, and although causing a short delay in mortality it did not prevent it [36].

Low NO bioavailability plays a role in murine CM pathogenesis [9] and it can help to explain the increased expression of eCAMs in brain vessels with the consequent rolling and adherence of leukocytes during PbA infection. Indeed, impaired NO production by NO synthase (NOS) inhibitors such as L-NAME, or by NOS gene knockout, has been shown to increase eCAM expression in vivo and in vitro [19-21,25]. Increased eCAM expression as well as vascular inflammation and platelet activation are also observed in hemolytic pathologies such as sickle cell crisis, in which cell-free hemoglobin acts as a potent NO scavenger limiting NO bioavailability [27]. Increased eCAM expression is followed by increased leukocyte rolling and adherence [25], and NO supplementation with NO donors can inhibit eCAM expression and leukocyte migration [26,40]. Here we show that exogenous NO indeed caused a significant downregulation of ICAM-1 and P-selectin, but not VCAM-1, expression in the brain of PbA-infected mice and, therefore, this may help to explain the protective effect of NO on murine CM. It is noteworthy, however, that exogenous NO decreased but did not actually prevent ICAM-1 expression, and did not significantly decrease VCAM-1 expression in PbA-infected mice. Accordingly, it decreased but did not prevent leukocyte and platelet accumulation in the brain on day 6, although it did prevent vascular leakage. While low NO bioavailability is an important component of endothelial dysfunction, it is not the sole cause of vascular inflammation in murine CM, which is the result of a complex cascade of events, including early activation of T cells [41] and production and release of high levels of several inflammatory cytokines [8]. The partial inhibitory effect of NO supplementation on the inflammatory process during PbA infection is in agreement with our previous study showing that exogenous NO supplementation with DPTA-NO attenuated but did not inhibit the brain microcirculatory complications in PbA infection [18].

In saline-treated PbA-infected mice, the venular sites with higher accumulation of leukocytes presented higher platelet counts. Because there were also direct correlations between the number of adherent leukocytes/platelets and venular diameters, this finding indicates that the differential quantitative accumulation was due basically to the larger available area for binding in larger vessels rather than to eventual differential expression of eCAMs in different vascular beds, although the latter was not directly assessed and cannot be ruled out. This is in accordance with previous findings by Sun and colleagues regarding platelet adherence during murine CM [42]. These results indicate that, during PbA infection, cerebral venules of different sizes in the range analyzed are similarly activated and similarly receptive to leukocyte and platelet adherence. DPTA-NO treatment, however, seems to differentially affect venules according to their size, as it had little effect on leukocyte accumulation in smaller (20-30 μm) venules and marked effect in larger (30-50 μm) ones. This effect is important not only in the sense that decreased inflammation may help preserve vascular integrity, but also because it decreases leukocyte-induced increases in vascular resistance, therefore increasing venous return and potentially improving blood flow. In our previous study [18], DPTA-NO sustained superior hemodynamics compared to saline, which in part is due to reduced vascular resistance due to geometrical and inflammatory changes. The results of these studies strongly support the view that NO therapy with a stable and long-acting molecule may constitute a useful therapeutic approach to partially decrease receptor-ligands interactions involved in CM cell adhesion and related hemodynamic complications. This may be true as well for mediators such as erythropoietin, which enhances endothelium-dependent vasodilatation mediated by NO in rodent cerebral vessels [43] and prevents vascular inflammation [44] and has also been shown to decrease inflammatory markers and partially protect mice against CM development [45].

Several studies have shown that murine CM is associated with BBB breakdown with consequent vascular leakage [29,35]. Here we studied the vascular leakage at the level of individual pial blood vessels and show that arterioles and venules were similarly affected in PbA-infected mice leading to increased albumin leakage to the brain tissue. Because no correlation was found between leakage and leukocyte/platelet adherence or vessel diameters in venules, and because leakage occurred in arterioles where leukocyte/platelet adherence was minimal, these results indicate that the endothelial dysfunction leading to increased permeability in the brain vessels is not dependent on close contact with inflammatory cells. In our study in no case the studied sites presented evidence of vessel wall rupture, which would lead to massive leakage and hemorrhage. Therefore, although no correlation was found between sites of leakage and of leukocyte/platelet accumulation, we were not able to establish any relation between the latter and more severe vascular damage. Inhibition of NO production can result in increased vascular permeability [46] and therefore the low NO bioavailability during PbA infection can help to explain increased vascular leakage. Exogenous NO treatment was indeed sufficient to prevent vascular leakage in pial vessels. This is again in accordance with our previous data showing marked protection against brain hemorrhages provided by DPTA-NO treatment, despite only partial effect on pial hemodynamics [18].

In summary, exogenous NO supplementation significantly decreases brain vascular inflammation during PbA infection by decreasing the expression of eCAMs and consequently decreasing recruitment of leukocytes and platelets, which eventually results in decreased vascular dysfunction and damage.

## Competing interests

The authors declare that they have no competing interests.

## Authors' contributions

GMZ infected and treated the mice and performed the eCAM expression studies, analyzed and interpreted data and wrote the manuscript; PC performed the intravital microscopy studies, analyzed and interpreted data and performed statistical analysis; WB participated in the intravital microscopy experiments; JAF analyzed and interpreted data; LJMC designed research, analyzed and interpreted data and wrote the manuscript. All authors have read and approved the final version of this manuscript.
